# Visualization of droplet spread produced by a nebulizer during the COVID-19 pandemic

**DOI:** 10.1093/qjmed/hcab169

**Published:** 2021-06-17

**Authors:** H Kato, T Ohya, Y Arai, K Nakagawa

**Affiliations:** 1 Infection Prevention and Control Department, Yokohama City University Hospital, 3-9 Fukuura, Kanazawa-ku, Yokohama 236-0004, Japan; 2 Department of Oral and Maxillofacial Surgery, Yokohama City University Graduate School of Medicine, 3-9 Fukuura, Kanazawa-ku, Yokohama 236-0004, Japan; 3 Department of Otolaryngology, Head and Neck Surgery, Yokohama City University School of Medicine, 3-9 Fukuura, Kanazawa-ku, Yokohama 236-0004, Japan; 4 Department of Bioengineering, Department of Precision Engineering, The University of Tokyo, 7-3-1 Hongo, Bunkyo-ku, Tokyo 113-8656, Japan

The nosocomial transmission of severe acute respiratory syndrome coronavirus 2 (SARS-CoV-2) in many medical settings has been widely reported.^1^ The droplet and airborne transmission are regarded as major routes of the transmission of SARS-CoV-2. Nebulizer therapy is considered risky during the coronavirus disease 2019 (COVID-19) pandemic due to its production of droplets^2^; however, droplet production by a nebulizer remains unclear.

Nebulizer therapy was introduced by Barach and colleagues^3^ in 1946 as a treatment for bronchitis and bronchiectasis. The effectiveness of nebulizer therapy was recently demonstrated for treating sinusitis and subglottic laryngitis^4^; as a result, nebulizer therapy was approved to treat these diseases by medical insurance companies and widely performed in in-hospital and outpatient settings in Japan. Generally, nebulizers are placed in a room without active ventilation, and the interval between use by different patients is usually 5 min or less. The safety of using nebulizers during the COVID-19 pandemic remains controversial.^5^ A nebulizer produces aerosol. Moreover, many patients who undergo nebulizer therapy have underlying diseases, such as sinusitis and bronchial asthma, and tend to cough by the stimulation of nebulizer use.

In our study, we recruited a healthy volunteer to test droplet production by a nebulizer. Written informed consent was taken. Our study was approved by the Yokohama City University Hospital review board (approval number: B200800048). The volunteer was asked to sit at a desk facing a nebulizer. Acrylic walls were placed around the subject to simulate a room where nebulizer therapy occurred and enable the observation of the droplets. The subject was confirmed to have no symptoms of upper respiratory infections before the observation. During the experiment, he was asked to wear eye protection to avoid damage by laser. SARS-CoV-2 is contained in droplets of 2.5 µm and larger in diameter.^1^ Thus, Particle Viewer PV2-VLD (Katokoken, Isehara, Japan), which enabled the observation of droplets of 3 µm and larger, was used. A summary of the system is available (https://www.youtube.com/watch?v=PlApulRMH9I, in Japanese). A red laser sheet at 638 nm was irradiated vertically from 2 m from the front of the subject to his right acromion. A green laser sheet at 532 nm was simultaneously irradiated horizontally at the level of the subject’s nipples. A video camera was set at 60 frames per second to photograph for 10 s, then at 1 frame per second for 300 s.

A commercially available, jet-type, aerosol-generating nebulizer (Millicon Pro, Shin-Ei Industries, Inc., Tokyo, Japan) was used.^6^ The Millicon Pro system generates particles with a median aerodynamic mass diameter of 4.6 µm. When the nebulizer, filled with 1% cefmenoxime (20 mg/2 ml), 0.1% dexamethasone (2 mg/2 ml) and 0.1% epinephrine (1 mg/1 ml), was activated, the subject inhaled by mouth through a suction hose and mouse piece from the nebulizer. Then the subject was instructed to inhale twice from the nebulizer normally holding a nebulizer.

**Figure 1.**
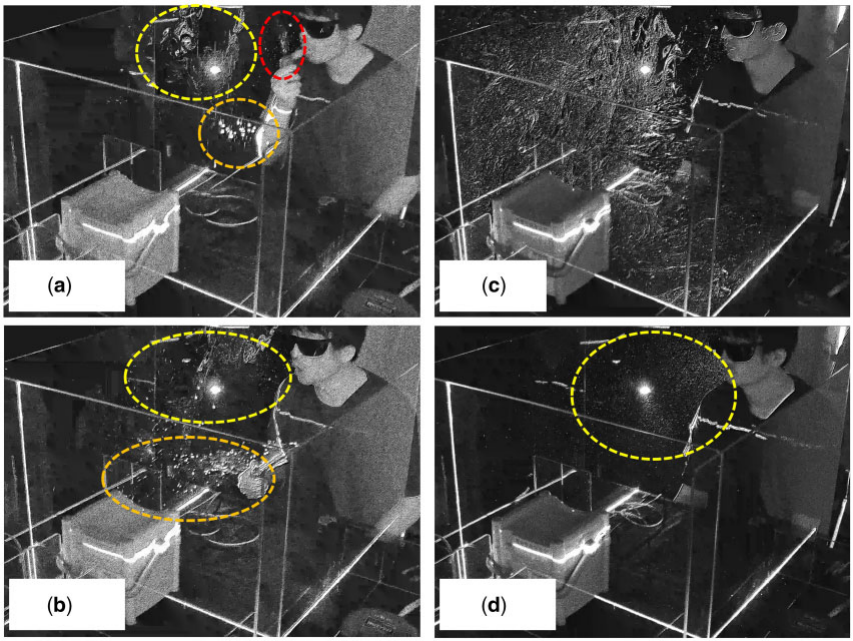
Droplets flew parabola (orange-coloured dotted line) and fine droplets flew upward from the nebulizer (red arrow) when the subject was taking normal breath-holding nebulizer (**a**). When the subject coughed, droplets passed straight forward (orange-coloured dotted line) (**b**), and fine droplets produced by cough kept flowing (yellow-coloured dotted line) for 30 s (**c**) and 300 s (**d**) after coughing.

When the subject was taking a normal breath while holding the nebulizer (Figure 1a), droplets were observed to flow upward (red dotted line circle) and flew parabola (orange-coloured dotted line) from the nebulizer. Fine droplets were also observed floating, filling the space enclosed by the acrylic walls. When the subject coughed, droplets were observed to pass straight forward (orange-coloured dotted line) (Figure 1b). Moreover, the cough-induced fine droplets were observed to continue to flow (yellow-coloured dotted line) for 30 and 300 s after coughing (Figure 1c and d).

It is difficult to diagnose SARS-CoV-2 infected patients based on symptoms because almost 80% of the infected persons are asymptomatic or have very mild symptoms.^7^ Thus, nebulizer use has been controversial during the COVID-19 pandemic. Our observations have two significant implications. First, active ventilation should be performed with nebulizer therapy to reduce the risk of nosocomial transmission. Second, once a patient uses a nebulizer in a room without active ventilation, the air in the room is contaminated for 300 s or more; using the nebulizer during this interval is not safe for the next patient.
